# Distinctive subgroups derived by cluster analysis based on pain and psychological symptoms in Swedish older adults with chronic pain – a population study (PainS65+)

**DOI:** 10.1186/s12877-017-0591-4

**Published:** 2017-09-02

**Authors:** Britt Larsson, Björn Gerdle, Lars Bernfort, Lars-Åke Levin, Elena Dragioti

**Affiliations:** 1 Pain and Rehabilitation Centre and Department of Medical and Health Sciences (IMH), Faculty of Health Sciences, Linköping University, 581 85 Linköping, SE Sweden; 2 Division of Health Care Analysis and Department of Medical and Health Sciences, Linköping University, 581 85 Linköping, SE Sweden

**Keywords:** Chronic pain, Older adults, Sub group analysis, Quality of life, Health care costs, Psychological distress

## Abstract

**Background:**

Improved knowledge based on clinical features of chronic pain in older adults would be valuable in terms of patient-orientated approaches and would provide support for health care systems in optimizing health care resources. This study identifies subgroups based on pain and psychological symptoms among Swedish older adults in the general population and compares derived subgroups with respect to socio-demographics, health aspects, and health care costs.

**Methods:**

This cross-sectional study uses data collected from four registers and one survey. The total sample comprised 2415 individuals ≥65 years old. A two-step cluster analysis was performed. Data on pain intensity, number of pain sites, anxiety, depression, and pain catastrophizing were used as classification variables. Differences in socio-demographics, quality of life, general health, insomnia, and health care costs among the clusters were investigated. Association of the clusters with the above parameters was further evaluated using multinomial logistic regression.

**Results:**

Four major clusters were identified: Subgroup 1 (*n* = 325; 15%) – moderate pain and high psychological symptoms; Subgroup 2 (*n* = 516; 22%) – high pain and moderate psychological symptoms; Subgroup 3 (*n* = 686; 30%) – low pain and moderate psychological symptoms; and Subgroup 4 (*n* = 767; 33%) – low pain and low psychological symptoms. Significant differences were found between the four clusters with regard to age, sex, educational level, family status, quality of life, general health, insomnia, and health care costs. The multinomial logistic regression analysis revealed that Subgroups 1 and 2, compared to Subgroup 4, were significantly associated with decreased quality of life, decreased general health, and increased insomnia. Subgroup 3, compared to Subgroup 4, was associated with decreased general health and increased insomnia. In addition, compared to Subgroup 4, Subgroups 1 and 2 were significantly associated with higher health care costs.

**Conclusions:**

Two high risk clusters of older adults suffering from chronic pain; one mainly based on psychological symptoms and one mainly on pain intensity and pain spread, associated with decreased quality of life and health and increased health care costs were identified. Our findings indicate that subgroup-specific treatment will improve pain management and reduce health care costs.

**Electronic supplementary material:**

The online version of this article (10.1186/s12877-017-0591-4) contains supplementary material, which is available to authorized users.

## Background

Chronic pain in older adults is a major public health problem worldwide. The prevalence of chronic pain in individuals aged ≥65 years ranges from 24% to 72% [1–5]. Chronic pain in this age group has substantial impact on pain-related disability [5–7], quality of life [6, 8, 9], depression [6, 10], sleep, and pain-related mobility [2, 6, 10]. In addition, chronic pain has considerable impacts on health care costs [8, 9, 11–13]; a recent study showed that the economic burden among older adults is high and related to the intensity of pain and age [9].

When managing chronic pain management, duration and frequency of pain must be considered [1, 14–18]. Another emerging issue, which may amplify the disability in the elderly, is the extent of spreading of pain [15, 16, 18–21]. Furthermore, psychological symptoms (e.g., anxiety, depression, and catastrophizing) are crucial parts in pain-related disability and suffering [4, 22–27]. These psychological symptoms might have larger effects on disability and quality of life than pain itself [23–25, 28, 29]. Taken together, these studies indicate that chronic pain is difficult to assess and to manage [30], so this complex situation needs to be understood if the elderly are to receive the best health care possible [6, 10, 30, 31]. Often undiscovered and untreated chronic pain in elderly [6, 10, 32, 33] is related to pain being misattributed to the natural ageing process [6], cognitive decline making assessing pain difficult [10, 32], other chronic conditions receiving more attention and care, and reluctance of physicians to prescribe pain medication due to high risk of adverse side effects [33].

Identification of homogeneous subgroups of elderly based on clinically important features – e.g., pain characteristics and psychological symptoms – would have practical value in terms of patient-orientated approaches [22, 34, 35] and would provide support for health care systems by helping these systems optimize resources and costs [36]. Identifying subgroups can be done by clustering individuals according to their pain symptoms. Clustering is particularly of interest in conditions that have high prevalence, burden, and costs [37]. Several cluster studies regarding pain, mainly in cohorts of patients, have been performed, but only a few studies have focused on older adults [34, 38]. Hence few studies have used cluster classification to examine this population for the combination of pain aspects, such as pain intensity and pain spreading on the body, and psychological symptoms.

Based on the literature, we hypothesized that for elderly individuals in the general population chronic pain together with prominent psychological strain is associated with a life situation worse than having chronic pain together with low psychological strain. To this end, we identified subgroups of individuals ≥65 years old based on pain characteristics and psychological distress and compared these subgroups with respect to age, sex, education, family status, health aspects, and costs.

## Methods

### Participants and procedure

**Fig. 1:**
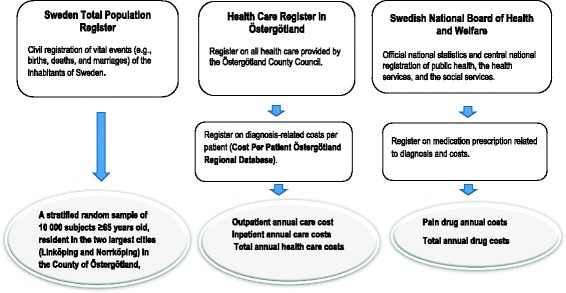
Registers and information retrieved from the registers

This study collected data from one survey and four registers. The survey was a cross-sectional postal questionnaire used to collect data from a stratified random sample of 10,000 older adults (individuals aged ≥65 years old) based on five age strata (65 to 69 years, 70 to 74 years, 75 to 79 years, 80 to 84 years, and 85 years and older) from the Swedish Total Population Register for the two largest cities (Linköping and Norrköping) of the County of Östergötland (south-eastern Sweden). The questionnaire was mailed in October 2012; if needed, as many as two postal reminders were mailed at two-week intervals. The collection of questionnaires closed in January 2013. In addition to the Swedish Total Population Register, this study also used data from the Health Care Register in Östergötland, the regional Cost Per Patient database, and a drug prescription register from the National Board of Health and Welfare (Fig. 1).

### Measurements

The survey was composed from several validated instruments. The relevant instruments for this study are described below. An overview of all parts of the survey has been presented elsewhere [9].

### Demographic aspects

Age, sex, educational level, and family status were recorded from the respondents’ answers in the postal survey.

### Characteristics of pain

We identified potential participants using three predetermined responses to the question “Do you usually have pain, either all the time or occasionally?”: 1) no; 2) yes, with less duration than 3 months; or 3) yes, with duration of more than 3 months. Only responders who marked 3 were considered eligible to participate in this study while the responders who marked 1 and 2 were excluded. For the responders who marked 3, their pain intensity was also registered using a numeric rating scale (NRS) for the previous 7 days (NRS7d) with the end points 0 (no pain) to 10 (worst imaginable pain) [17, 39]. Next, the responders identified the spreading of pain on a drawing with 45 predefined sites on a body manikin that included the number and location of the pain they had identified using the NRS for pain during previous 7 days [16]. Hence, the number of pain sites ranged between 1 and 45; high values indicated higher spreading of pain.

### General health, anxiety, and depression

The General Well Being Scale (GWBS) [40] consists of 18 items on psychological well-being and distress. The first 14 questions use a six-point rating scale (ranging from 0 to 5) that represents intensity or frequency, and the remaining four items use an 11-point rating scale with the end points 0 (very concerned) and 10 (not concerned at all). The instrument has provided good internal consistency, test–retest reliability, and validity [40]. From the GWBS, six subscales can be calculated [41]: GWBS-General health – Items 10, 15; possible range – items 0–15; GWBS-Anxiety – items 2, 5, 8, 16; possible range – items 0–25; GWBS-Depression – items 4, 12, 18; possible range – items 0–20. In addition, subscales on positive well-being, self- control, and vitality can be calculated. This study presents data from the subscales for general health, anxiety, and depression. For the subscales of anxiety and depression, low values indicate higher anxiety and depression; the items of these subscales were reverse scored so high values indicate higher symptoms of anxiety and depression.

### The pain Catastrophizing scale

The Pain Catastrophizing Scale (PCS) measures three dimensions of catastrophizing – rumination, magnification, and helplessness – based on 13 items with five answer alternatives: 0 = “not at all”; 1 = “to a slight degree”; 2 = “to a moderate degree”; 3 = “to a great degree”; and 4 = “all the time” [42]. The scale has adequate to excellent internal consistency and validity [43]. We summed the 13 items – the total PCS (PCS-total), so the maximum score was 52 –the higher score, the more catastrophizing [44]. Due to a technical failure, the most negative alternative (“all the time”) was not included in the questionnaire, so our PCS-total had possible scores between 0 and 39. However, we estimated the reliability of the instrument by calculating the Cronbach alpha (a) and it was found good (a = 0.75).

### The European quality of life 5 dimensions questionnaire instrument

The European Quality of Life Instrument 5 Dimensions (EQ-5D) captures perceived health-related quality of life (HRQL) as defined by five dimensions: mobility, self-care, usual activities, pain/discomfort, and anxiety/depression [45]. Each dimension has three possible levels (1, 2, or 3), representing “no problems”, “some problems”, and “extreme problems”, respectively. An individual index was calculated (EQ-5D Index) based on these five dimensions and a table based on the United Kingdom (UK) time trade-off scores for a subset of 45 EQ-5D HRQL states [45, 46]. This table displays the extent of problems on each of the five dimensions represented by a set of five-digit descriptors based on the combination of the three possible levels (e.g., 11,121, 33,211, etc.) and each of those combinations of digit descriptors corresponds to an individual index value (e.g., 11,121 = 0.796, 33,211 = 0.086, etc.). The index ranges from - 0.594 to 1. To measure self-estimation of current HRQL, we used the European Quality of Life Vertical Visual Analogue (EQ-VAS), a standard vertical 100-point thermometer-like scale with defined end points “Best imaginable health state” and “Worst imaginable health state”. High values indicate good HRQL and low values indicate bad HRQL [46].

### Insomnia severity index

The Insomnia Severity Index (ISI) is a reliable and valid instrument for quantifying insomnia severity [47, 48]. The seven items of ISI are rated on a 5-point Likert scale (0–4). The instrument yields a total score range between 0 and 28. The total score can be divided into four categories: no clinically significant insomnia (ISI: 0–7); sub-threshold insomnia (ISI: 8–14); moderate clinical insomnia (ISI: 15–21); and severe clinical insomnia (ISI: 22–28). In this study, only the summed score of ISI was used. The ISI has provided excellent psychometric properties [47, 48].

### Health care costs

Data on inpatient (hospital days and treatments) and outpatient (visits to various categories of health care providers) care were retrieved from the Health Care Register in Östergötland (Fig. 1). To determine costs of health care consumption, data from the Health Care Register in Östergötland were linked to the regional Cost Per Patient database provided by Östergötland County Council. Drug prescription data were retrieved from the National Board of Health and Welfare. These registers have been described in detail elsewhere [9].

Information on all health care and medicine costs related to the participants in our study were gathered by matching the national personal identification numbers to the above registers. The following costs were included in the present study: outpatient care, inpatient care, total costs for all medicine outlets, total health care costs, and total costs for pain drugs.

### Statistics

All statistical analyses were performed using SPSS version 22.0 for Windows (IBM Corp., Armonk, NY, USA). All tests were two-tailed and statistical significance was defined as a value of *p* ≤ 0.05. Data are presented as mean ± standard deviation (SD) for continuous variables and as count (percentage) for categorical variables.

In a first step, we aimed to identify clusters (i.e., subpopulations of Swedish older adults with chronic pain) based on the clinical symptoms of pain intensity, number of pain sites, anxiety, depression, and pain catastrophizing. Hence, these variables were considered as classification variables. To this end, a two-step cluster analysis (TSCA) was performed. In the TSCA, the number of clusters was determined automatically by first running pre-clustering and then by hierarchical methods on basis of the best fit. The classification variables were also z-standardized by default in order to be commensurable (Additional file 1). Assumption of the independence between the classifications variables were also examined (Additional file 1: Table S2). TSCA has several proper features that distinguish it from traditional clustering techniques (i.e., hierarchical and K -mean). These features make it possible to define clusters based on both categorical and continuous variables to be analysed from large data sets, to specify the importance of predictor variables included in the analysis, and to automatically select the number of clusters. Furthermore, TSCA is more effective for continuous variables when the sample is large (>200) [49]. Model fit was assessed by Schwarz’s Bayesian information criterion (BIC) and evaluated by the average silhouette coefficient, which evaluates cluster cohesion and separation measures*.* The BIC is a criterion for model selection among a finite set of models; the model with the lowest BIC is preferred [50]. The silhouette coefficient is an internal validity index that typically ranges between 0 and 1: the closer to 1, the better the model. The smallest BIC value accompanied by the highest values of BIC change, (i.e., the ratio of BIC changes and the ratio of distances) and an average silhouette coefficient equal or above 0.50 indicates good model fit [51]. Next, we investigated the derived clusters with respect to age, sex, education, family status, quality of life, general health, insomnia, and health care costs (all continuous denominated external variables) using the Chi-square test for categorical variables and one-way analysis of variance (ANOVA) for continuous variables. When significant differences were found (*p* ≤ 0.05), we performed bivariate multinomial logistic regression analysis to identify possible significant associations of the clusters with the above-mentioned external variables, presented as odds ratios (OR) with 95% confidence intervals (CIs). Multinomial logistic regression is an extension of binary logistic regression and it is used when the dependent variable has more than two nominal categories. Hence, our dependent variable had four possible categories (Subgroup 1, Subgroup 2, Subgroup 3, and Subgroup 4). We chose Subgroup 4 as the baseline reference category, to assess the odds of Subgroup 1 vs Subgroup 4, the odds of Subgroup 2 vs Subgroup 4, and the odds of Subgroup 3 vs Subgroup 4. Since we explored natural subpopulations (i.e., clusters), we did not control for covariates in the multinomial logistic regression analysis.

## Results

### General characteristics of the total sample

**Fig. 2:**
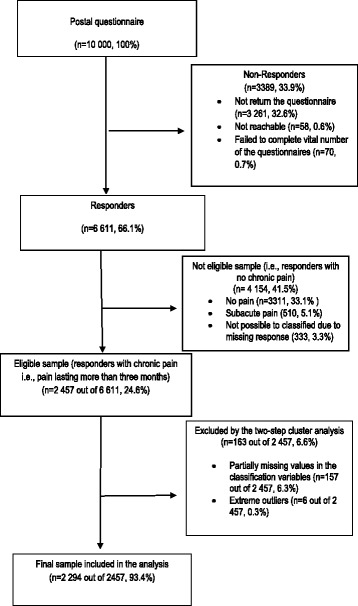
Flow chart showing study population from initial sample to final eligible sample

Of the 10,000 subjects selected from the sample, 3261 did not return the questionnaire, 58 were not reachable, and 70 failed to complete vital number of the questionnaires, leaving a total of 6611 (66.1%) valid responses. Of these, 4154 respondents were further excluded because they did not meet the criteria for chronic pain (i.e., pain lasting more than 3 months) (Fig. 2). Therefore, the total sample consisted of 2457 older adults – 981 men (39.9%) and 1476 women (60.1%) – with chronic pain. The mean age was 75.9 (SD = 7.4) and there was no significant difference in age between the men and women. The comparison between baseline characteristics of the subjects included with those excluded from the study revealed that there were significant differences in the sex (*p* < 0.001) and education distribution (*p* = 0.003), indicating that those who excluded were more likely to be men and higher educated compared to those included. The additional comparison analysis between the subjects excluded due to missing data and those included in the analysis showed significant differences, indicating that those with missing data were more likely to be in older ages (*p* < 0.001), women (*p* = 0.002), secondary educated (*p* = 0.024) and widowed (*p* = 0.011) relative to those included in the analysis.

The mean values of the classification variables in the whole sample were pain intensity (M = 5.0; SD = 2.0), number of pain sites (5.7; SD = 5.2), anxiety (M = 6.3; SD = 4.8), depression (M = 5.5; SD = 3.7), and pain catastrophizing (M = 11.8; SD = 8.6) (Table 1).

**Table 1:** Classification variables of the total sample, the clusters (subgroups) and overall comparisons among the subgroups

Variables** (Mean ± SD; otherwise stated)	Total sample	Subgroup 1*	Subgroup 2^†^	Subgroup 3^‡^	Subgroup 4^§^	*p* value^a^
Total number of participants (n, *%*)	2.294 (100.0)	325 (14.6)	516 (22.1)	686 (29.9)	767 (33.4)	
Pain intensity (NRS7d), range 0–10	5.00 ± 2.0	6.5 ± 1.8	7.4 ± 1.9	4.1 ± 1.2	4.0 ± 1.5	<0.001
Number of pain sites, range 1–45	5.7 ± 5.2	6.7 ± 5.0	11.5 ± 6.9	3.8 ± 2.3	3.9 ± 2.7	<0.001
GWBS-Anxiety, range 0–25	6.3 ± 4.8	13.5 ± 3.8	6.10 ± 3.2	7.5 ± 3.3	1.9 ± 1.7	<0.001
GWBS- Depression, range 0–20	5.5 ± 3.7	10.6 ± 3.2	5.2 ± 2.6	6.8 ± 2.4	2.1 ± 1.4	<0.001
PCS, range 0–39	11.8 ± 8.6	24.1 ± 7.3	13.6 ± 7.5	10.2 ± 7.4	7.5 ± 4.9	<0.001

*GWBS* General Well-being Schedule, *NRS7d* Pain intensity as measured by a numeric rating scale for the previous 7 days, *PCS* Pain catastrophizing scale, *Subgroup 1 = older adults with moderate pain and high psychological symptoms; ^†^Subgroup 2 = older adults with high pain and moderate psychological symptoms; ^‡^Subgroup 3 = older adults with low pain and moderate psychological symptoms; ^§^Subgroup 4 = older adults with low pain without psychological symptoms; ^a^One way ANOVA; SD = standard deviation. **For all classification variables high values indicate worsening health status

### The two-step cluster analysis

The analysis excluded 163 individuals: 157 due to partially missing values in the questionnaires regarding the classification variables and six due to extreme values in those variables (i.e., outliers). As a result, the analysis included a valid number of 2294 out of 2457 (93%) individuals with chronic pain to be clustered and further analysed (Fig. 2).

Four major clusters (Subgroups 1–4) based on pain intensity, number of pain sites, anxiety, depression, and pain catastrophizing were identified (Table 1). Although the lowest BIC coefficient was for nine clusters, the optimal number of clusters was four, because the largest BIC change, the ratio of BIC changes and the ratio of distances, were for four clusters (data not shown). Additionally, the quality of the model for the four clusters was good: average silhouette coefficient = 0.50, indicating a reasonable cluster structure [51]. The most important predictors of cluster/subgroup membership among the classification variables were depression and anxiety (both predictor importance = 1). However, in order to evaluate the stability of the cluster solution, a hierarchical cluster analysis was also performed in a random sample of 50% of the participants, and identical results were observed as those in the two step cluster analysis. On the other random 50% we performed K-means cluster methods and got the same results.

### Description of the derived clusters

Table 1 shows the distribution of the four clusters and the comparisons among them. The description of the clusters is based on the mean values of each classification variable in relation to the mean values of those variables in the whole sample (Table 1). Thus, the mean values of the classification variables one unit or more above the mean values of the total sample were considered high. Mean values differing less than one unit or more from the mean values of the total sample were considered moderate. Mean values one unit or lower than the mean values of the total sample were considered low.

Subgroup1 (*n* = 325; 15%) had high mean values for pain intensity, moderate mean values for number of pain sites, and high mean values for anxiety, depression, and pain catastrophizing. Subgroup 2 (*n* = 516; 22%) had the high mean values for pain intensity and number of pain sites, moderate mean values for anxiety and depression, and high mean values for pain catastrophizing. Subgroup 3 (*n* = 686; 30%) had low mean values for pain intensity and number of pain sites and moderate mean values for anxiety, depression, and pain catastrophizing. Subgroup 4 (*n* = 767; 33%) had low mean values in all the classification variables (Table 1). Significant overall differences with regard to classification variables were found among the four subgroups (*p* < 0.001) (Table 1).

### Comparisons between subgroups with respect to external variables

In the univariate analysis, overall significant differences were found between the four subgroups with regard to sociodemographic aspects (i.e., age, sex, education, and family status) (all *p* < 0.001) (Table 2). Significant differences between the four subgroups were also found with respect to quality of life (i.e., EQ-5D-index and EQ-5D-VAS), general health, insomnia, and annual all health care costs (all *p* < 0.001) (Table 3).

**Table 2:** Mean values (±SD) of socio-demographic characteristics in the four subgroups and comparisons among the subgroups

Variables	Subgroup 1* (*n* = 325)	Subgroup 2^†^ (*n* = 516)	Subgroup 3^‡^ (*n* = 686)	Subgroup 4^§^ (*n* = 767)	*p* value^a^
Age (years)^b^	77.8 ± 7.7	76.0 ± 7.4	76.2 ± 7.3	73.1 ± 6.7	<0.001
Sex (%)					<0.001
Men	36.3	36.2	39.7	45.0	
Women	63.7	63.8	60.3	55.0	
Education (%)					<0.001
High school (secondary)	61.5	54.8	54.5	49.1	
Upper secondary school or vocational training ≥2 years	23.7	26.0	25.7	26.9	
College or university for ≤2 years	5.7	10.1	7.6	9.3	
College or university ≥3 years	9.1	9.1	12.2	14.8	
Family status (%)					<0.001
Married	47.4	53.7	58.3	61.8	
Single	3.7	6.0	4.7	5.3	
Divorced	19.7	17.1	14.4	15.1	
Widowed	29.2	25.3	22.6	17.7	

*Subgroup 1 = older adults with moderate pain and high psychological symptoms; ^†^Subgroup 2 = older adults with high pain and moderate psychological symptoms; ^‡^Subgroup 3 = older adults with low pain and moderate psychological symptoms; ^§^Subgroup 4 = older adults with low pain without psychological symptoms; ^a^One way ANOVA for continuous variables and chi square test for categorical variables; ^b^Mean ± SD = standard deviation

**Table 3:** External variables concerning health aspects and costs in the subgroups and comparisons among the subgroups

Variables (mean ± SD)	Subgroup 1* (*n* = 325)	Subgroup 2^†^ (*n* = 516)	Subgroup 3^‡^ (*n* = 686)	Subgroup 4^§^ (*n* = 767)	*p* value^a^
EQ-5D-index	0.41 ± 0.4	0.56 ± 0.3	0.69 ± 0.2	0.76 ± 0.1	<0.001
EQ-5D-VAS (0–100)	45.9 ± 21.0	57.2 ± 21.6	64.3 ± 19.9	75.9 ± 17.2	<0.001
GWBS general health	5.1 ± 3.1	8.7 ± 5.1	9.8 ± 4.6	12.3 ± 2.1	<0.001
Insomnia	14.4 ± 5.2	11.7 ± 4.9	10.5 ± 4.5	7.5 ± 4.3	<0.001
Outpatient annual care cost (Euros)	2937 ± 3948	2586 ± 3580	2002 ± 3163	1751 ± 3335	<0.001
Inpatient annual care cost (Euros)	2368 ± 7948	1778 ± 6901	1395 ± 5975	878 ± 3782	<0.001
Pain drug annual cost (Euros)	116 ± 303	101 ± 236	35 ± 103	25 ± 88	<0.001
Total annual drug cost (Euros)	890 ± 1462	733 ± 1410	510 ± 942	406 ± 913	<0.001
Total annual health care cost (Euros)	6200 ± 10,405	5098 ± 9170	3907 ± 8526	3030 ± 6014	<0.001

EQ-5Dindex = The European quality of life instrument; EQ-5D-VAS (0–100) = The European quality of life instrument thermometer-like scale, GWBS = General Well-being Schedule; *Subgroup 1 = older adults with moderate pain and high psychological symptoms; ^†^Subgroup 2 = older adults with high pain and moderate psychological symptoms; ^‡^Subgroup 3 = older adults with low pain and moderate psychological symptoms; ^§^Subgroup 4 = older adults with low pain without psychological symptoms; ^a^One way ANOVA; SD = standard deviation

The results of the multinomial logistic regression analysis are presented in Table 4. Compared to Subgroup 4, both Subgroup 1 and Subgroup 2 were associated with decreased quality of life according to the EQ-5D-index (Subgroup 1: OR = 0.02, 95% CI: 0.01–0.03, Subgroup 2: OR = 0.02, 95% CI: 0.01–0.10) and the EQ-5D-VAS (Subgroup 1: OR = 0.85, 95% CI: 0.67–0.99, Subgroup 2: OR = 0.98, 95% CI: 0.97–0.99) (Table 4). A similar pattern was found for general health. Compared to Subgroup 4, both Subgroup 1 and Subgroup 2 were also associated with increased levels of insomnia (OR = 1.23, 95% CI:1.17–1.28 and OR = 1.13, 95% CI: 1.08–1.17, respectively) and increased health care costs (i.e., outpatient care, inpatient care, total costs for all medicine outlets, total health care, and total costs for pain medicine) (Table 4) and Subgroup 3 were associated with decreased general health (OR = 0.66, 95% CI: 0.62–0.72) and increased levels of insomnia (OR = 1.10, 95% CI: 1.07–1.14) (Table 4). Sex, age, educational level, and family status were not significant variables in the multinomial logistic regression analysis, although the univariate analysis revealed significant differences between the four clusters with respect to those variables (Table 2).

**Table 4:** Bivariate multinomial logistic regression analysis of factors associated with the clusters/subgroups (*n* = 2294)

	Clusters								
	Subgroup 1* vs subgroup 4^§^			Subgroup 2^†^ vs subgroup 4^§^			Subgroup 3^‡^ vs subgroup 4^§^		
Variables	OR	95% CI	*p* value	OR	95% CI	*p* value	OR	95% CI	*p* value
Age (years)	1.02	(0.98–1.07)	0.203	1.00	(0.97–1.04)	0.750	1.02	(0.99–1.05)	0.144
Men (Reference group)	1.00	-	-	1.00	-	-	1.00	-	-
Women	1.29	(0.72–2.32)	0.390	1.22	(0.77–1.93)	0.383	0.90	(0.61–1.34)	0.627
High school (secondary) (Reference group)	1.00	-	-	1.00	-	-	1.00	-	-
Upper secondary school or vocational training ≥2 years	0.78	(0.39–1.56)	0.490	1.17	(0.68–1.99)	0.558	1.08	(0.67–1.73)	0.745
College or university for ≤2 years	0.31	(0.09–1.05)	0.060	1.34	(0.66–2.75)	0.413	0.93	(0.48–1.81)	0.846
College or university ≥3 years	0.85	(0.34–2.16)	0.748	0.65	(0.31–1.37)	0.266	0.86	(0.47–1.57)	0.630
Married (Reference group)	1.00	-	-	1.00	-	-	1.00	-	-
Single	1.34	(0.35–5.14)	0.661	1.53	(0.61–3.92)	0.366	1.05	(0.43–2.61)	0.903
Divorced	1.92	(0.91–4.01)	0.082	0.93	(0.51–1.72)	0.829	1.17	(0.69–1.98)	0.557
Widowed	1.23	(0.57–2.63)	0.594	1.07	(0.58–1.98)	0.827	0.96	(0.54–1.68)	0.888
EQ-5D-index	0.02	(0.01–0.03)	**<0.001**	0.02	(0.01–0.10)	**<0.001**	0.30	(0.06–2.24)	0.281
EQ-5D-VAS (0–100)	0.85	(0.67–0.99)	**0.022**	0.98	(0.97–0.99)	**<0.001**	0.99	(0.99–1.01)	0.874
GWBS general health	0.45	(0.56–0.68)	**<0.001**	0.71	(0.61–0.82)	**<0.001**	0.66	(0.62–0.72)	**<0.001**
Insomnia	1.23	(1.17–1.28)	**<0.001**	1.13	(1.08–1.17)	**<0.001**	1.10	(1.07–1.14)	**<0.001**
Outpatient annual care cost (Euros)	1.10	(1.02–1.07)	**0.008**	1.06	(1.01–1.12)	**0.029**	1.00	(1.00–1.00)	0.132
Inpatient annual care cost (Euros)	1.11	(1.01–1.05)	**0.002**	1.02	(1.01–1.03)	**0.002**	1.00	(1.00–1.00)	0.084
Pain drug annual cost (Euros)	1.06	(1.01–1.04)	**0.050**	1.02	(1.01–1.03)	**0.040**	0.99	(0.99–1.01)	0.777
Total annual drug cost (Euros)	1.05	(1.01–1.13)	**0.041**	1.02	(1.01–1.04)	**0.050**	1.00	(1.00–1.00)	0.579
Total annual health care cost (Euros)	1.03	(1.01–1.02)	**0.050**	1.01	(1.01–1.02)	**0.050**	0.99	(0.99–1.00)	0.843

EQ-5Dindex = The European quality of life instrument; EQ-5D-VAS (0–100) = The European quality of life instrument thermometer-like scale, GWBS = General Well-being Schedule; OR = odds ratio; CI = confidence interval

*Subgroup 1 = older adults with moderate pain and high psychological symptoms; ^†^Subgroup 2 = older adults with high pain and moderate psychological symptoms; ^‡^Subgroup 3 = older adults with low pain and moderate psychological symptoms; ^§^Subgroup 4 = older adults with low pain without psychological symptoms; Reference category = ^§^Subgroup 4; Nagelkerke R^2^ = 0.36; all significant factors (*p* ≤ 0.05) in bolds

## Discussion

There were three main findings of this population study of older adults with chronic pain:

- We found four clusters based on pain intensity, spreading of pain on the body, anxiety, depression, and pain catastrophizing in older adults. In particular, we identified two high risk clusters (Subgroups 1 and 2) comprising one-third of the study sample and two less affected clusters (Subgroups 3 and 4) in terms of symptom severity.
- Subgroup 1 was mainly described by increased psychological symptoms and Subgroup 2 was mainly described by increased pain intensity and pain spreading.
- The multinomial logistic regression analysis revealed that, compared to Subgroup 4, Subgroups 1 and 2 were associated with decreased quality of life, decreased general health, increased insomnia, and increased health care costs and Subgroup 3 was associated with decreased general health and increased insomnia.

The age range in this population study of older adults and the classification variables used make it difficult to compare the results with previous research. Nevertheless, our results are supported by previous studies of patients, including older adults, with chronic pain and fibromyalgia syndrome, [34–36, 38, 52, 53]. Four of those studies [34–36, 52] found at least one subgroup of patients with moderate pain intensity and elevated psychological symptoms similar to our Subgroup 1 and one subgroup with low levels of pain intensity and psychological symptoms (i.e., similar to Subgroup 4). Additionally and in line with our results, one of these studies found one subgroup of individuals with pain the previous 4 weeks (> 50 years old) had on average ten pain sites, corresponding to our Subgroup 2, and another subgroup reported on average five pain sites, corresponding to our Subgroup 1 [38].

One main finding consistent with previous clustering studies was the documentation of psychological symptoms as variables that differentiate chronic pain clusters [34–36, 52]. Thus, we found that that the identification of Subgroup1 was mainly based on high levels of depression, anxiety, and pain catastrophizing. It is well known that depression, anxiety, and pain catastrophizing are common in cohorts of individuals with chronic pain [22, 34–36, 52]. Subgrouping studies not specifically focused on older adults have found a variety of clusters in cohorts with chronic pain based on those psychological symptoms [22, 23, 35, 52]. For example, Loevinger et al. [52] found at least three subgroups of chronic pain patients with high psychological symptoms. The question may arise how the reported values of anxiety and depression relate to clinical anxiety and/or depression. The GWBS instrument - including the subscales of depression and anxiety - was chosen since it is designed for epidemiological studies. To the best of our knowledge there exist no clinical cut off values for the subscales of GWBS indicating definite increased risk for clinical depression or anxiety. Hence, in future studies it will be important to determine if e.g. the levels of anxiety and/or depression in subgroup 2 (Table 1) represent clinical anxiety and/or depression or subclinical levels.

There was a predominance of elevated pain intensity and number of pain sites, representing spreading of pain on the body in Subgroup 2, distinguishing this subgroup from the other subgroups. The impact of pain spreading in cluster analysis/methodology of pain conditions is in accordance with findings reported from previous cross-sectional studies [38, 54]. One of those studies reported significance of pain spreading for derivation of clusters in elderly individuals [38]. That study, unlike our study, did not examine the significance of pain intensity and of psychological symptoms for the cluster formation [38]. Evidence of spreading of pain as a substantial pain characteristic is growing [16, 19, 21, 55], especially since a strong dose-response relationship between spreading of pain and work disability has been found [19]. Furthermore, spreading of pain on the body has been shown to be associated with pain frequency and duration [16], with chronic diseases in the general population [16, 55], and with poorer lower extremity function [15]. Moreover, spreading of pain on the body increases the risk of frailty in older populations [21]. There is also emerging evidence that pain intensity, as in this study of older individuals with chronic pain, is another important pain characteristic that can be used to identify clinically relevant subgroups [14].

Mean age, the proportion of men and women, educational level, and family status were not similar across the four subgroups. That is, Subgroups 1 and 2 were older, had lower proportions of men, were less educated, and were more often not married (Table 2). Similarly, Hirsh et al. [36] found that demographic characteristics are associated with clusters of individuals suffering from pain. However, in our more precise multinomial logistic regression analysis, we were unable to confirm such sociodemographic dependences (Table 4).

According to the multinomial logistic regression analysis, markedly poor quality of life and general health were associated with Subgroup 1 (characterized by moderate pain and high psychological symptoms). Insomnia was also higher in this subgroup, compared to the other subgroups, with an increased odd ratio of almost 23%. The second highest affected subgroup in terms of quality of life and general health was Subgroup 2 (characterized by high pain level and moderate psychological symptoms). Moreover, for Subgroups 1 and 2 an impaired quality of life was found as well as Subgroups 1, 2, and 3 were associated with decreased general health although the strongest association was found in Subgroup 1. These findings could mean that two distinct mechanisms are responsible for decreased health-related quality of life in older adults with chronic pain. One mechanism could be based on depression, anxiety, and catastrophic beliefs about pain and one could be based on severity of pain (i.e., spreading of pain and intensity of pain). Indeed, several studies suggest that psychological symptoms may have more influence on pain-related consequences than pain itself [23–25, 28, 29]. From the other side of the spectrum, both pain intensity and spreading are now considered as potentially important indicators of low health-related quality of life in older adults with chronic pain [9, 20]. For example, Lacey et al. [20] in a large population-based survey of musculoskeletal pain in individuals ≥50 years old found a dose-response relationship between the extent of pain on the body and quality of life. These results match those observed in earlier studies that have found that psychological comorbidities could contribute to the link between health-related factors, insomnia, and chronic pain [2, 5, 7, 8, 10].

Other studies have found that a relatively small proportion of those with chronic pain consume the majority of the resources [56, 57]. The multinomial logistic regression analysis revealed that Subgroups 1 and 2 were associated with elevated health care costs (all five investigated variables). This finding, to some extent, agrees with Hirsh et al. [36]. They found at least one subgroup of patients with chronic severe pain and comorbid depression (similar to our Subgroup1) was associated with the highest health care costs [36]. Our study agrees with a recent systematic review that was not restricted to older adults [58]; that review reported increased direct health care costs in chronic low back pain with mental disorders, mainly depression.

Considering individuals with chronic pain as a homogenous group, as often done in clinical trials, may yield debatable clinical outcomes [59]. Thus, multicomponent approaches including psychological treatments might be appropriate for individuals corresponding to Subgroup1, whereas more pain targeting treatment might be suitable for individuals corresponding to Subgroup 2. This relationship could indicate that health care costs will be reduced when specific treatment efforts are made with respect to these two subgroups.

This study is the first of its kind to identify pain clusters in older adults by taking into account core symptomatology of chronic pain. The large sample size is another strength, giving us good power for the reported findings. However, this study has some limitations. The cross sectional study design is unable to identify directions of casualty. Another limitation is that we used post surveys instead of a face-to-face clinical examination, which has been shown to be associated with more robust assessments [3], although we used valid instruments with reliable psychometric properties to measure the presence of the studied symptomatology. Even though pain intensity and spreading of pain were found to be important aspects for the formation of the subgroups factors such as pain interference may also predict outcomes in this field. In future investigations of subgroups in patients with chronic this dimensions should be included. The fact that PCS due to a technical mistake (the most negative alternative of each item was not included) had a shorter range of responses decreases the variability of PCS and might be associated with an underestimation of the importance of PCS when identifying the four subgroups. This study does not consider the role of physical comorbidities and cognitive functioning, factors that may be associated with the chronic pain in older adults [3, 6, 18, 30]. Additional research should be undertaken in this age group that includes these factors in order to develop clinically valid and easily detectable subgroups. Finally, the two step cluster analysis is easily accessible, but also ambiguous mainly because it is based on a “distance-based cluster method” instead of a probabilistic modelling approach (such as latent class analysis; LCA). The main difference is that LCA is a “top-down approach” which better describes the distribution of the data and provides information on the classification probabilities for individual classification while the other clustering algorithms are rather “bottom-up approaches” which are based on the dissimilarities or similarities between cases. The two step cluster, however, uses a similar method to LCA to choose the optimal subgroup model, and it has been found to perform regularly better than traditional hierarchical cluster techniques (i.e., hierarchical and K -mean) [60].

## Conclusions

In conclusion, there were at least two high-risk subgroups of individuals comprising approximately one-third of the studied population of older adults with severe implications for individuals, health care providers, and the health care system. One of the most important and clinically relevant findings was the significance of pain intensity and pain spreading on the body along with psychological symptoms in generating subgroups in a Swedish general population. The presence of significant subgroups of patients with chronic pain have been described in highly selected groups of chronic pain patients e.g. at clinical departments of University hospitals [61, 62]. An important clinical aspect of the present study is that such subgroups are present in the population and a broad assessment including psychological aspects are necessary when patients are seeking primary health care. Furthermore, increased health care costs were associated with either prominent pain or prominent psychological symptoms. Identifying and treating these two subgroups based on the actual pattern of symptoms has the potential to improve health and quality of life and decrease health care costs; clearly, more studies on this topic are needed.

## Additional file

Additional file 1: Results of normality tests and bivariate correlations of the classification variables. **Table S1.** Tests of normality with the use of Kolmogorov-Smirnov and Shapiro-Wilk tests. **Figure S1.** Q-Q plots of raw data (1st figure) and z scores (2nd figure) of pain intensity. **Figure S2.** Q-Q plots of raw data (1st figure) and z scores (2nd figure) of spreading of pain. **Figure S3.** Q-Q plots of raw data (1st figure) and z scores (2nd figure) of anxiety. **Figure S4.** Q-Q plots of raw data (1st figure) and z scores (2nd figure) of depression. **Figure S5.** Q-Q plots of raw data (1st figure) and z scores (2nd figure) of pain catastrophizing. **Table S2.** Bivariate correlations of the classifications variables. (DOCX 227 kb)

## Abbreviations

BIC: Bayesian information criterion
EQ-5D: European Quality of Life Instrument 5 Dimensions
EQ-VAS: European Quality of Life Vertical Visual Analogue
GWBS: General Well Being Scale
ISI: Insomnia Severity Index
OR: Odds ratio
PCS: Pain Catastrophizing Scale
TSCA: two-step cluster analysis

## References

[1] Brattberg G, Parker MG, Thorslund M (1996). The prevalence of pain among the oldest old in Sweden. Pain.

[2] Covinsky KE, Lindquist K, Dunlop DD, Yelin E (2009). Pain, functional limitations, and aging. J Am Geriatr Soc.

[3] Helme RD, Gibson SJ (2001). The epidemiology of pain in elderly people. Clin Geriatr Med.

[4] Jakobsson U, Klevsgard R, Westergren A, Hallberg IR (2003). Old people in pain: a comparative study. J Pain Symptom Manag.

[5] Patel KV, Guralnik JM, Dansie EJ, Turk DC (2013). Prevalence and impact of pain among older adults in the United States: findings from the 2011 National Health and aging trends study. Pain.

[6] AGS Panel on Persistent Pain in Older Persons. The management of persistent pain in older persons. J Am Geriatr Soc. 2002;50(6 Suppl):S205–24. 10.1046/j.1532-5415.50.6s.1.x 12067390

[7] Raftery MN, Sarma K, Murphy AW, De la Harpe D, Normand C, McGuire BE (2011). Chronic pain in the Republic of Ireland--community prevalence, psychosocial profile and predictors of pain-related disability: results from the prevalence, impact and cost of chronic pain (PRIME) study, part 1. Pain.

[8] Breivik H, Collett B, Ventafridda V, Cohen R, Gallacher D (2006). Survey of chronic pain in Europe: prevalence, impact on daily life, and treatment. Eur J Pain.

[9] Bernfort L, Gerdle B, Rahmqvist M, Husberg M, Levin LA (2015). Severity of chronic pain in an elderly population in Sweden--impact on costs and quality of life. Pain.

[10] Molton IR, Terrill AL (2014). Overview of persistent pain in older adults. Am Psychol.

[11] Andrew R, Derry S, Taylor RS, Straube S, Phillips CJ (2014). The costs and consequences of adequately managed chronic non-cancer pain and chronic neuropathic pain. Pain Pract.

[12] Doherty E, O'Neill C (2014). Estimating the health-care usage associated with osteoarthritis and rheumatoid arthritis in an older adult population in Ireland. J Public Health.

[13] Stockbridge EL, Suzuki S, Pagan JA (2015). Chronic pain and health care spending: an analysis of longitudinal data from the medical expenditure panel survey. Health Serv Res.

[14] Bromley Milton M, Borsbo B, Rovner G, Lundgren-Nilsson A, Stibrant-Sunnerhagen K, Gerdle B (2013). Is pain intensity really that important to assess in chronic pain patients? A study based on the Swedish quality registry for pain rehabilitation (SQRP). PLoS One.

[15] Eggermont LH, Bean JF, Guralnik JM, Leveille SG (2009). Comparing pain severity versus pain location in the MOBILIZE Boston study: chronic pain and lower extremity function. J Gerontol A Biol Sci Med Sci.

[16] Grimby-Ekman A, Gerdle B, Bjork J, Larsson B (2015). Comorbidities, intensity, frequency and duration of pain, daily functioning and health care seeking in local, regional, and widespread pain - a descriptive population-based survey (SwePain). BMC Musculoskelet Disord.

[17] Dunn KM, Jordan KP, Croft PR (2011). Contributions of prognostic factors for poor outcome in primary care low back pain patients. Eur J Pain.

[18] Croft P, Jordan K (2005). Jinks C: "pain elsewhere" and the impact of knee pain in older people. Arthritis Rheum.

[19] Kamaleri Y, Natvig B, Ihlebaek CM, Bruusgaard D (2009). Does the number of musculoskeletal pain sites predict work disability? A 14-year prospective study. Eur J Pain.

[20] Lacey RJ, Belcher J, Rathod T, Wilkie R, Thomas E, McBeth J (2014). Pain at multiple body sites and health-related quality of life in older adults: results from the north Staffordshire osteoarthritis project. Rheumatology.

[21] Wade KF, Lee DM, McBeth J, Ravindrarajah R, Gielen E, Pye SR, Vanderschueren D, Pendleton N, Finn JD, Bartfai G (2016). Chronic widespread pain is associated with worsening frailty in European men. Age Ageing.

[22] Hill JC, Fritz JM (2011). Psychosocial influences on low back pain, disability, and response to treatment. Phys Ther.

[23] Leeuw M, Goossens ME, Linton SJ, Crombez G, Boersma K, Vlaeyen JW (2007). The fear-avoidance model of musculoskeletal pain: current state of scientific evidence. J Behav Med.

[24] Parmelee PA, Katz IR, Lawton MP (1991). The relation of pain to depression among institutionalized aged. J Gerontol.

[25] Pincus T, Burton AK, Vogel S, Field AP (2002). A systematic review of psychological factors as predictors of chronicity/disability in prospective cohorts of low back pain. Spine.

[26] Quartana PJ, Campbell CM, Edwards RR (2009). Pain catastrophizing: a critical review. Expert Rev Neurother.

[27] Sullivan MJ, Reesor K, Mikail S, Fisher R (1992). The treatment of depression in chronic low back pain: review and recommendations. Pain.

[28] Crombez G, Vlaeyen JW, Heuts PH, Lysens R (1999). Pain-related fear is more disabling than pain itself: evidence on the role of pain-related fear in chronic back pain disability. Pain.

[29] Lame IE, Peters ML, Vlaeyen JW, Kleef M, Patijn J (2005). Quality of life in chronic pain is more associated with beliefs about pain, than with pain intensity. Eur J Pain.

[30] Vellucci R (2012). Heterogeneity of chronic pain. Clin Drug Invest.

[31] Karoly P, Ruehlman LS (2006). Psychological "resilience" and its correlates in chronic pain: findings from a national community sample. Pain.

[32] Landi F, Onder G, Cesari M, Gambassi G, Steel K, Russo A, Lattanzio F, Bernabei R (2001). Pain management in frail, community-living elderly patients. Arch Intern Med.

[33] Gianni W, Ceci M, Bustacchini S, Corsonello A, Abbatecola AM, Brancati AM, Assisi A, Scuteri A, Cipriani L, Lattanzio F (2009). Opioids for the treatment of chronic non-cancer pain in older people. Drugs Aging.

[34] Hall-Lord ML, Larsson G, Steen B (1999). Chronic pain and distress in older people: a cluster analysis. Int J Nurs Pract.

[35] Wilson HD, Robinson JP, Turk DC (2009). Toward the identification of symptom patterns in people with fibromyalgia. Arthritis Rheum.

[36] Hirsch O, Strauch K, Held H, Redaelli M, Chenot JF, Leonhardt C, Keller S, Baum E, Pfingsten M, Hildebrandt J (2014). Low back pain patient subgroups in primary care: pain characteristics, psychosocial determinants, and health care utilization. Clin J Pain.

[37] Lim SS, Vos T, Flaxman AD, Danaei G, Shibuya K, Adair-Rohani H, Amann M, Anderson HR, Andrews KG, Aryee M (2012). A comparative risk assessment of burden of disease and injury attributable to 67 risk factors and risk factor clusters in 21 regions, 1990-2010: a systematic analysis for the global burden of disease study 2010. Lancet.

[38] Lacey RJ, Strauss VY, Rathod T, Belcher J, Croft PR, Natvig B, Wilkie R, McBeth J (2015). Clustering of pain and its associations with health in people aged 50 years and older: cross-sectional results from the north Staffordshire osteoarthritis project. BMJ Open.

[39] Ferreira-Valente MA, Pais-Ribeiro JL, Jensen MP (2011). Validity of four pain intensity rating scales. Pain.

[40] Fazio AF. A concurrent validational study of the NCHS general well-being schedule. Vital Health Stat 2. 1977;(73):1–53. PMID:610049. 610049

[41] McDowell I. Measuring health: a guide to rating scales and questionnaires. USA Oxford University Press; 2006.

[42] Sullivan MJL, Bishop SR, Pivik J (1995). The pain catastrophizing scale: development and validation. Psychol Assess.

[43] Osman A, Barrios FX, Kopper BA, Hauptmann W, Jones J, O'Neill E (1997). Factor structure, reliability, and validity of the pain Catastrophizing scale. J Behav Med.

[44] Miro J, Nieto R, Huguet A (2008). The Catalan version of the pain Catastrophizing scale: a useful instrument to assess catastrophic thinking in whiplash patients. J Pain.

[45] Greiner W, Weijnen T, Nieuwenhuizen M, Oppe S, Badia X, Busschbach J, Buxton M, Dolan P, Kind P, Krabbe P (2003). A single European currency for EQ-5D health states. Results from a six-country study. Eur J Health Econ.

[46] Dolan P, Sutton M (1997). Mapping visual analogue scale health state valuations onto standard gamble and time trade-off values. Soc Sci Med.

[47] Bastien CH, Vallieres A, Morin CM (2001). Validation of the insomnia severity index as an outcome measure for insomnia research. Sleep Med.

[48] Morin CM, Belleville G, Belanger L, Ivers H (2011). The insomnia severity index: psychometric indicators to detect insomnia cases and evaluate treatment response. Sleep.

[49] Cluster analysis from Statnotes: Topics in Multivariate analysis [http://faculty.chass.ncsu.edu/garson/pa765/statnote.htm]. Accessed 20 May 2016.

[50] Gideon S (1978). Estimating the dimension of a model. Ann Stat.

[51] Kaufman L, Rousseeuw PJ. Finding groups in data: an introduction to cluster analysis. New York: Wiley; 1990.

[52] Loevinger BL, Shirtcliff EA, Muller D, Alonso C, Coe CL (2012). Delineating psychological and biomedical profiles in a heterogeneous fibromyalgia population using cluster analysis. Clin Rheumatol.

[53] de Souza JB, Goffaux P, Julien N, Potvin S, Charest J, Marchand S (2009). Fibromyalgia subgroups: profiling distinct subgroups using the fibromyalgia impact questionnaire. A preliminary study. Rheumatol Int.

[54] Schmidt CO, Baumeister SE (2007). Simple patterns behind complex spatial pain reporting? Assessing a classification of multisite pain reporting in the general population. Pain.

[55] Mundal I, Grawe RW, Bjorngaard JH, Linaker OM, Fors EA (2014). Prevalence and long-term predictors of persistent chronic widespread pain in the general population in an 11-year prospective study: the HUNT study. BMC Musculoskelet Disord.

[56] Linton SJ, Hellsing AL, Hallden K (1998). A population-based study of spinal pain among 35-45-year-old individuals. Prevalence, sick leave, and health care use. Spine.

[57] Linton SJ, Ryberg M (2000). Do epidemiological results replicate? The prevalence and health-economic consequences of neck and back pain in the general population. Eur J Pain.

[58] Baumeister H, Knecht A, Hutter N (2012). Direct and indirect costs in persons with chronic back pain and comorbid mental disorders--a systematic review. J Psychosom Res.

[59] Hazard RG, Spratt KF, McDonough CM, Olson CM, Ossen ES, Hartmann EM, Carlson RJ, LaVoie J (2012). Patient-centered evaluation of outcomes from rehabilitation for chronic disabling spinal disorders: the impact of personal goal achievement on patient satisfaction. Spine J.

[60] Kent P, Jensen RK, Kongsted A (2014). A comparison of three clustering methods for finding subgroups in MRI, SMS or clinical data: SPSS TwoStep cluster analysis, Latent Gold and SNOB. BMC Med Res Methodol.

[61] Denison E, Asenlof P, Sandborgh M, Lindberg P (2007). Musculoskeletal pain in primary health care: subgroups based on pain intensity, disability, self-efficacy, and fear-avoidance variables. J Pain.

[62] Giesecke T, Williams DA, Harris RE, Cupps TR, Tian X, Tian TX, Gracely RH, Clauw DJ (2003). Subgrouping of fibromyalgia patients on the basis of pressure-pain thresholds and psychological factors. Arthritis Rheum.

