# Supplementary material for: Distinctive subgroups derived by cluster analysis based on pain and psychological symptoms in Swedish older adults with chronic pain – a population study (PainS65+)
Source: BMC Geriatr. 2017 Sep 2;17:200. doi: 10.1186/s12877-017-0591-4 (PMC5581449; doi:10.1186/s12877-017-0591-4)
Supplement: Additional file 1: — Results of normality tests and bivariate correlations of the classification variables. Table S1. Tests of normality with the use of Kolmogorov-Smirnov and Shapiro-Wilk tests. Figure S1. Q-Q plots of raw data (1st figure) and z scores (2nd figure) of pain intensity. Figure S2. Q-Q plots of raw data (1st figure) and z scores (2nd figure) of spreading of pain. Figure S3. Q-Q plots of raw data (1st figure) and z scores (2nd figure) of anxiety. Figure S4. Q-Q plots of raw data (1st figure) and z scores (2nd figure) of depression. Figure S5. Q-Q plots of raw data (1st figure) and z scores (2nd figure) of pain catastrophizing. Table S2. Bivariate correlations of the classifications variables. (DOCX 227 kb) [file 12877_2017_591_MOESM1_ESM.docx]

Additional file 1

| **Table S1. Tests of normality with the use of Kolmogorov-Smirnov and Shapiro-Wilk tests** | | | | | | |
| --- | --- | --- | --- | --- | --- | --- |
| **Tests of Normality** | | | | | | |
|  | Kolmogorov-Smirnov^a^ | | | Shapiro-Wilk | | |
|  | Statistic | df | Sig. | Statistic | df | Sig. |
| 1. Raw data |  |  |  |  |  |  |
| Pain intensity | .121 | 2294 | .000 | .967 | 2294 | .000 |
| Spreading of pain | .192 | 2294 | .000 | .754 | 2294 | .000 |
| GWBS Anxiety | .107 | 2294 | .000 | .934 | 2294 | .000 |
| GWBS Depression | .118 | 2294 | .000 | .949 | 2294 | .000 |
| Pain catastrophizing | .078 | 2294 | .000 | .958 | 2294 | .000 |
| 1. Z normalization |  |  |  |  |  |  |
| Pain intensity | .121 | 2294 | .000 | .967 | 2294 | .000 |
| Spreading of pain | .193 | 2294 | .000 | .778 | 2294 | .000 |
| GWBS Anxiety | .107 | 2294 | .000 | .934 | 2294 | .000 |
| GWBS Depression | .118 | 2294 | .000 | .949 | 2294 | .000 |
| Pain catastrophizing | .078 | 2294 | .000 | .958 | 2294 | .000 |
| a. Lilliefors Significance Correction | | | | | | |


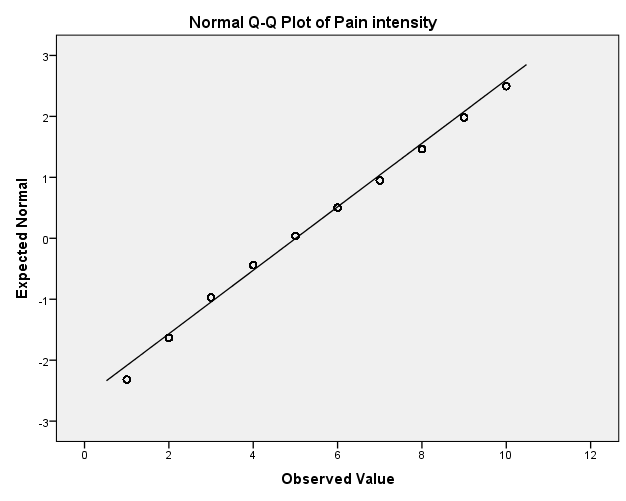


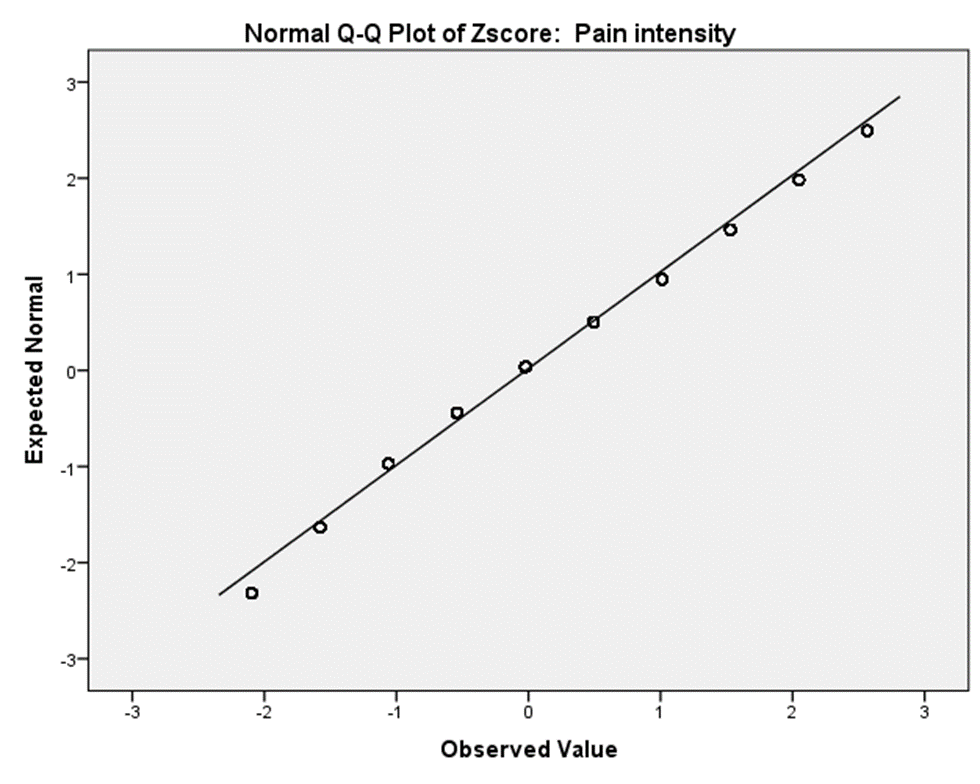


**Figure S1. Q-Q plots of raw data (1^st^ figure) and z scores (2^nd^ figure) of pain intensity**


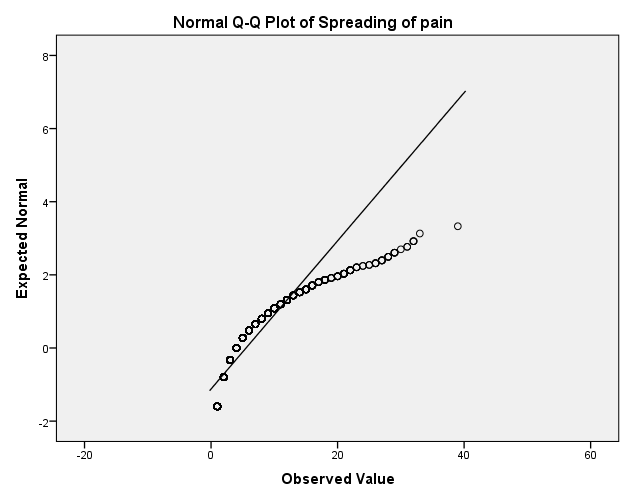


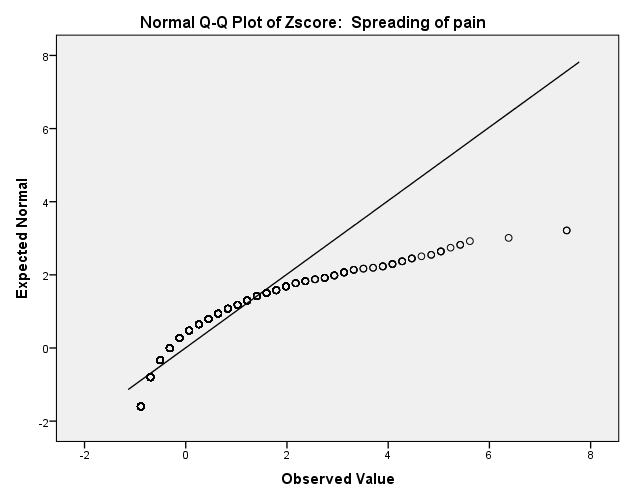


**Figure S2. Q-Q plots of raw data (1^st^ figure) and z scores (2^nd^ figure) of spreading of pain**


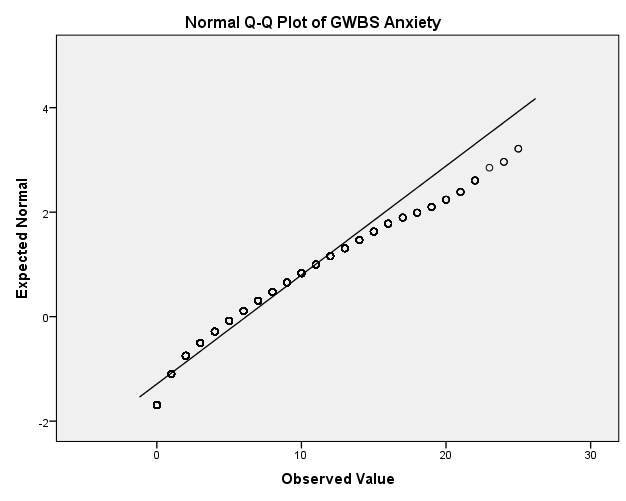


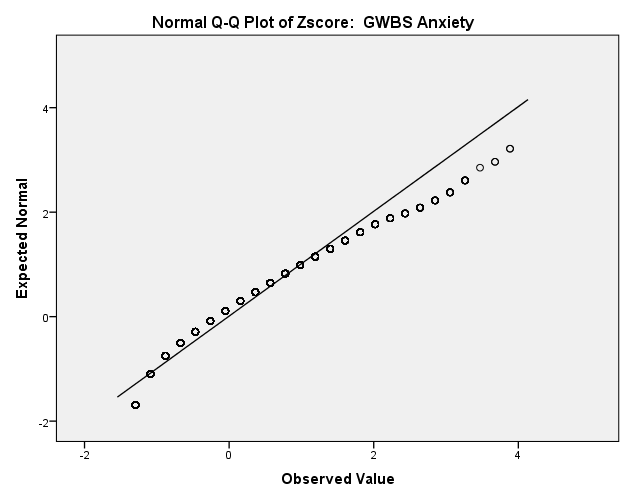


**Figure S3. Q-Q plots of raw data (1^st^ figure) and z scores (2^nd^ figure) of anxiety**


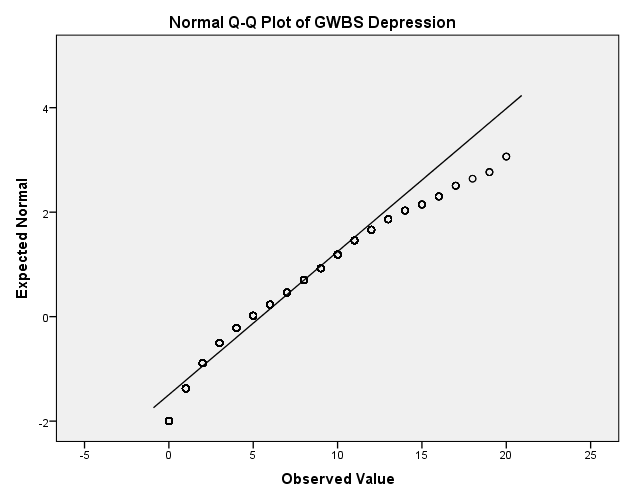

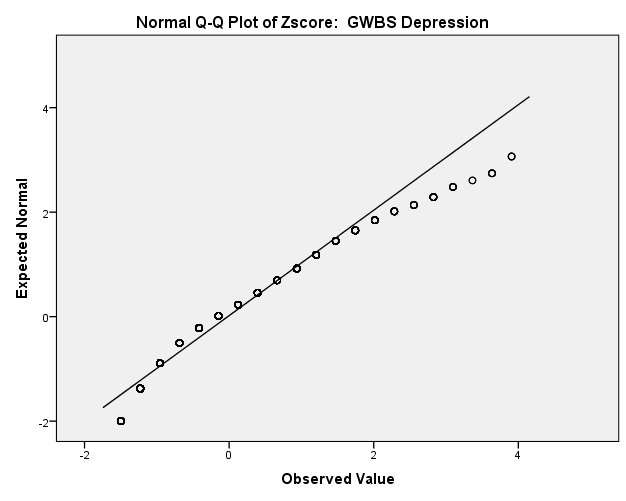


**Figure S4. Q-Q plots of raw data (1^st^ figure) and z scores (2^nd^ figure) of depression**


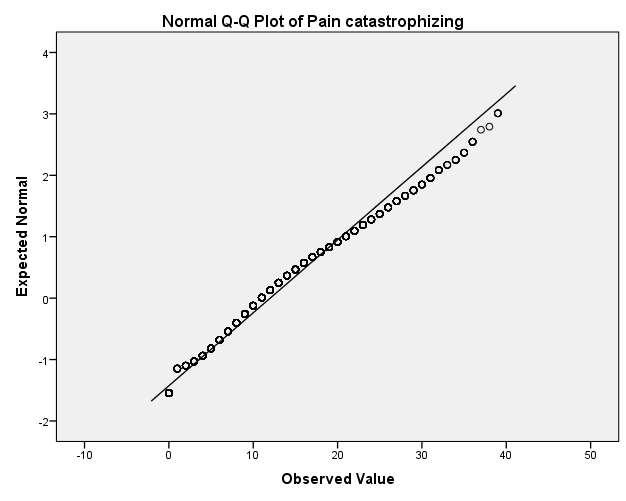


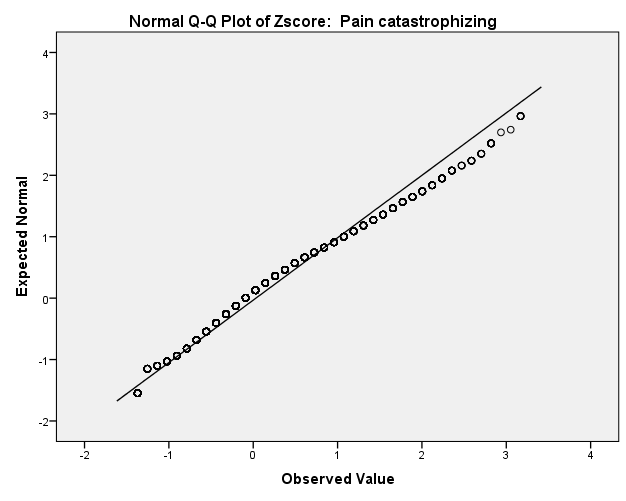


**Figure S5. Q-Q plots of raw data (1^st^ figure) and z scores (2^nd^ figure) of pain catastrophizing**

| **Table S2. Bivariate correlations of the classifications variables** | | | | | | | |
| --- | --- | --- | --- | --- | --- | --- | --- |
|  | Pain intensity | Spreading of pain | | GWBS Anxiety | GWBS Depression | Pain catastrophizing | |
| 1.Pain intensity | 1 |  |  | |  | |  |
| 2.Spreading of pain | .266^***^ | 1 |  | |  | |  |
| 3.GWBS Anxiety | .280^***^ | .196^***^ | 1 | |  | |  |
| 4.GWBS Depression | .218^***^ | .169^***^ | .519^***^ | | 1 | |  |
| 5.Pain catastrophizing | .348^***^ | .213^***^ | .422^***^ | | .364^***^ | | 1 |
| Notes: Pearson correlation coefficients are presented; ***p<0.001 | | | | | | | |
